# Sex-specific differences in ICOS^+^ T helper cell differentiation in systemic lupus erythematosus patients with low disease activity

**DOI:** 10.1007/s10238-024-01307-1

**Published:** 2024-03-01

**Authors:** Lisa Wu, Florian Kälble, Hanns-Martin Lorenz, Martin Zeier, Matthias Schaier, Andrea Steinborn

**Affiliations:** 1 Department of Obstetrics and Gynecology, University of Heidelberg, INF 440, 69120 Heidelberg, Germany; 2 Department of Nephrology, University of Heidelberg, INF 162, 69120 Heidelberg, Germany; 3 Department of Rheumatology, University of Heidelberg, INF 410, 69120 Heidelberg, Germany

**Keywords:** Systemic lupus erythematosus (SLE), Sex-specific CD4^+^ T cell differentiation, Inducible costimulatory molecule (ICOS), Responder T cells (Tresps), Recent thymic emigrants (RTEs), Resting mature naïve cells (MNs)

## Abstract

Systemic lupus erythematosus (SLE) is a sex biased chronic autoimmune disease affecting predominantly females during reproductive ages. Changes in the ratio of inducible costimulatory molecule (ICOS)^+^ regulatory (Treg) and non-regulatory responder (Tresp) CD4^+^ T cells proved to be crucial for the occurrence of high disease activity. Here, we investigated how the differentiation of ICOS^+^CD45RA^+^CD31^+^ recent thymic emigrant (RTE) Tresps into CD45RA^−^CD31^−^ memory Tresps affects the percentages of ICOS^+^ Tresps within total CD4^+^ T cells. Three different pathways (pathway 1 via CD45RA^−^CD31^+^ memory Tresps, pathway 2 via direct proliferation and pathway 3 via resting mature naïve CD45RA^+^CD31^−^ (MN) cells) were examined in healthy controls and SLE remission patients separated by sex. In female SLE remission patients, immunosuppressive therapy inhibited the ICOS^+^ RTE differentiation via CD45RA^−^CD31^+^ memory Tresps and direct proliferation, leaving an age-independently increased differentiation into CD45RA^−^CD31^−^ memory Tresps by conversion of resting MN Tresps compared with healthy controls. Due to exhaustion of this pathway with age, no age-dependent change in the percentages of ICOS^+^ Tresps within total CD4^+^ T cells could be found. In contrast, no age-independently increased differentiation could be detected in men due to sufficient immunosuppression of all three pathways. This allowed an age-dependent differentiation of ICOS^+^ RTE Tresps into CD45RA^−^CD31^−^ memory Tresps by conversion of resting MN Tresps, resulting in age-dependently increasing percentages of ICOS^+^ Tresps within total CD4^+^ T cells. We hypothesize that the sex-specific differential effect of immunosuppression on the differentiation of ICOS^+^ Tresps may explain the sex- and age-dependent occurrence of high disease activity.

**Supplementary Information:**

The online version contains supplementary material available at 10.1007/s10238-024-01307-1.

## Introduction

Systemic lupus erythematosus (SLE) is a chronic autoimmune disease characterized by a strong sex bias, with a much higher prevalence in women than in men. The overall female-to-male ratio is 9:1 but varies according to the geographical region and with age, with the highest ratio being observed around the female reproductive ages (20–40 years) [1–3]. For males, the peak of disease onset is later, in the fifth to seventh decade, leading to a decreased female-to-male ratio in that age group [4]. Although the background for these differences is not fully understood yet, studies support that the female predominance in SLE may be the result of a complex interaction between sex hormones, genetics, epigenetics, gut microbiota [5] and aging [6]. The fact that the female-to-male ratio is much lower in pre-pubertal-onset SLE (3.3:1) and increases in adolescent-onset SLE (7.25:1) supports the hypothesis of hormonal association with the demographic differences observed [7]. The disease is currently not curable and follows a course of remissions, under effective immunosuppressive therapy, and relapses [8]. It is very heterogeneous in its clinical presentation containing tissue injury in multiple organs, with lupus nephritis being the most common cause of morbidity and mortality [9]. While the disease mainly inflicts women, it could be observed that men diagnosed with SLE often have a more aggressive clinical course resulting in a poorer prognosis [10–12].

The pathophysiology of SLE includes strong hyperactivity of B and T cells, resulting in primarily non-organ-specific autoantibody production against nuclear self-antigens [13]. The immune pathogenesis involves many types of immune cells. However, CD4^+^ T cells seem to play a decisive role, since their characteristic decline and activation was ascertained in both SLE patients and lupus-prone mice [14]. Both numerical and functional disturbances have been reported in CD4^+^ T cell subsets, such as Th1, Th2, Th17, regulatory and follicular T helper cells [15]. For example, in SLE patients, a reduced number and function of regulatory T helper cells has been shown [16], while an increased frequency of Th17 cells and increased production of interleukin-17 was found [17, 18]. Furthermore, an uncontrolled expansion of Tfh cells was ascertained in lupus prone mice and SLE patients [19]. The proper differentiation of these CD4^+^ T cell subsets can be impaired by early signaling defects via cytokines and aberrant activation of kinases and phosphatases, leading to alterations in key metabolic pathways including glycolysis, glutaminolysis and oxidative phosphorylation [15]. Moreover, treatment of CD4^+^ T cells with mitochondrial metabolism inhibitors and glucose metabolism inhibitors reduced interferon-γ production and reversed disease biomarkers in lupus-prone mice [20].

For maintaining self-tolerance, particularly immunosuppressive regulatory CD4^+^ T cells (Tregs) are of great importance. Their dysfunction was shown to be crucial in the development of various autoimmune diseases including SLE [21, 22]. In addition, a possible resistance of non-regulatory responder CD4^+^ T cells (hereinafter referred as Tresps) to the suppressive effect of Tregs could contribute to pathogenesis, especially in lupus diseases [23–25].

Recent studies by our group showed differences in the differentiation of CD45RA^+^CD31^+^ recent thymic emigrant (RTE) Tregs and RTE Tresps in special conditions, such as pregnancy and renal insufficiency, compared to healthy controls. Increased differentiation of RTE Tregs/Tresps into CD45RA^−^CD31^−^ memory Tregs/Tresps (CD31^−^ memory Tregs/Tresps) was detected, with the differentiation pathway via CD45RA^−^CD31^+^ memory Tregs/Tresps (CD31^+^ memory Tregs/Tresps) or CD45RA^+^CD31^−^ mature naïve (MN) Tregs/Tresps influencing both the functional properties of Tregs/Tresps and the quantitative ratios in terms of their contribution to total CD4^+^ T cells [23, 26–29].

It is known that similar to pregnancy or renal insufficiency autoimmune diseases are associated with thymic involution, resulting in changes in the composition and function of the circulating T cell pool [30]. In diabetes-prone mice, defective thymic export of RTEs as well as non-productive post-thymic differentiation of RTEs have been shown, possibly enhancing the predisposition to develop autoimmune insulin-dependent diabetes mellitus [31].

For SLE patients, we distinguished between inducible costimulatory molecule (ICOS)^+^ and ICOS^−^ Tregs/Tresps [32, 33]. The smaller ICOS^+^ Treg and, presumably, ICOS^+^ Tresp population are known to be highly proliferative and more resistant to programmed cell death compared to the larger ICOS^−^ Treg/Tresp population [34], with ICOS being a known marker of T cell activation [35]. Studies with ICOS knockout mice [36] and ICOS deficient patients [37] have shown the importance of ICOS signaling for the development and effector function of different T cell subsets [38], indicating its crucial role in the regulation of immune responses in autoimmune diseases for both Tregs [39] and Tresps [40–42]. In SLE patients with positive anti-double-stranded DNA (anti-dsDNA) antibodies, significantly elevated frequencies of ICOS^+^ T cells have been shown (0.93% of total CD3^+^ T cells) compared to those negative for anti-dsDNA antibodies (0.27%, p < 0.05) and compared to healthy controls (0.08%, *p* < 0.001) [43]. This implies an increased activation of T cells in these patients but does not distinguish between different T cell subsets such as Tresps and immunosuppressive Tregs. Considering Tregs being only a minority of all T cells, these results presumably mostly represent Tresps. Previous studies by our group with mixed collectives of both sexes indicate that especially ICOS^+^ T cells play a role in the transition from low to high disease activity in SLE patients, as an age-independently increased ratio of ICOS^+^ Tregs/Tresps was shown in SLE patients in remission (median value 0.55, range 0.24–1.30) compared to healthy controls (median value 0.51, range 0.15–1.33; *p* < 0.05), but a decreased ratio in active SLE patients (mean value 0.38, range 0.14–0.72; *p* < 0.01) [32]. In this study, we examined shifts in the composition of the total CD4^+^ T helper cell pool with ICOS^+^ and ICOS^−^ Tregs/Tresps in patients with low disease activity (SLEDAI ≤ 7) separately for both sexes and focused on differences in the differentiation of especially ICOS^+^ Tresps between women and men.

## Materials and methods

### Patient collectives and healthy controls

Peripheral venous blood samples were collected from 300 healthy volunteers (Group 1: 150 women and 150 men) and 105 SLE patients with low disease activity (Group 2: 83 women and 22 men). All SLE patients fulfilled the 1983 revised and 1997 updated criteria of the American College of Rheumatology (ACR) for SLE. Patients were classified as in remission or with low disease activity if Systemic Lupus Erythematosus Disease Activity Index (SLEDAI) ≤ 7 (hereinafter referred to as remission). This cut-off value was established because our previous studies have shown that SLE patients with low disease activity, in whom disease activity is still largely suppressed by immunosuppression, show increased differentiation, whereas this differentiation is exaggerated and exhausted in patients in whom the disease becomes active. The relatively high cut-off value of 7 proved to be the most appropriate to differentiate between these two groups of patients. Of all SLE patients, 77% showed kidney involvement. Blood samples were collected during routine visits or during a hospital stay at the Department of Nephrology, University of Heidelberg.

### Positive selection of CD4^+^ T cells

Peripheral venous blood samples (9 ml) were collected from all participants into EDTA-containing tubes. CD4^+^ T cells were isolated by immune affinity chromatography with the FABian system (IBA GmbH, Göttingen, Germany), according to the manufacturer’s instructions. This cell isolation process is performed in the FABian column that is prefilled with a Strep-Tactin-Fab-anti-CD4-coated agarose matrix. The process starts with sucking up whole blood samples. CD4^+^ T cells bind to the matrix, while non-target cells are washed away. Adding D-biotin to the matrix causes a dissociation of Fab and target cells from the beads so that the CD4^+^ T cells can be recovered. Subsequently, the isolated CD4^+^ T cells were analyzed using six-color flow cytometry.

### Fluorescence-activated cell sorting (FACS) staining

Briefly, at most 8 × 10^6^ CD4^+^ T cells were surface stained with 5 µl peridinin-chlorophyll-protein-cyanine 5.5 (PerCpCy5.5)-conjugated anti-CD127 (clone eBioRDR5, eBioscience, Frankfurt, Germany), 20 µl phycoerythrin (PE)-conjugated anti-ICOS (clone DX29, BD Biosciences, Heidelberg, Germany), 5 µl allophycocyanin-H7 (APC-H7)-conjugated anti-CD45RA (clone HI100, BD Biosciences) and 5 µl phycoerythrin–cyanine 7 (PE-Cy7)-conjugated anti-CD31 (clone WM-59, eBioscience) mouse monoclonal antibodies. Intracellular staining for the detection of FoxP3 was performed using a fluorescein isothiocyanate (FITC)-conjugated anti-human FoxP3 staining set (clone PCH101, eBioscience) according to the manufacturer’s instructions. Detection of Ki67^+^ cells within the different Tresp subsets was performed by incubating the fixed cells with 2 µl Alexa Fluor 647-conjugated anti-Ki67 mouse monoclonal antibodies (clone B56, BD Biosciences). Negative control samples were incubated with isotype-matched antibodies. For doublet discrimination FSC-A versus FSC-H gating was used. To identify and exclude dead cells FSC-A versus SSC-A gating was applied. Cells were analyzed by a FACS Canto flow cytometer (BD Biosciences). Statistical analysis was based on at least 100.000 CD4^+^ T cells. The gating strategy for the presentation of the different CD4^+^ T cell subsets is given in Supplementary Fig. S1.

### Statistical analysis

**Fig. 1:**
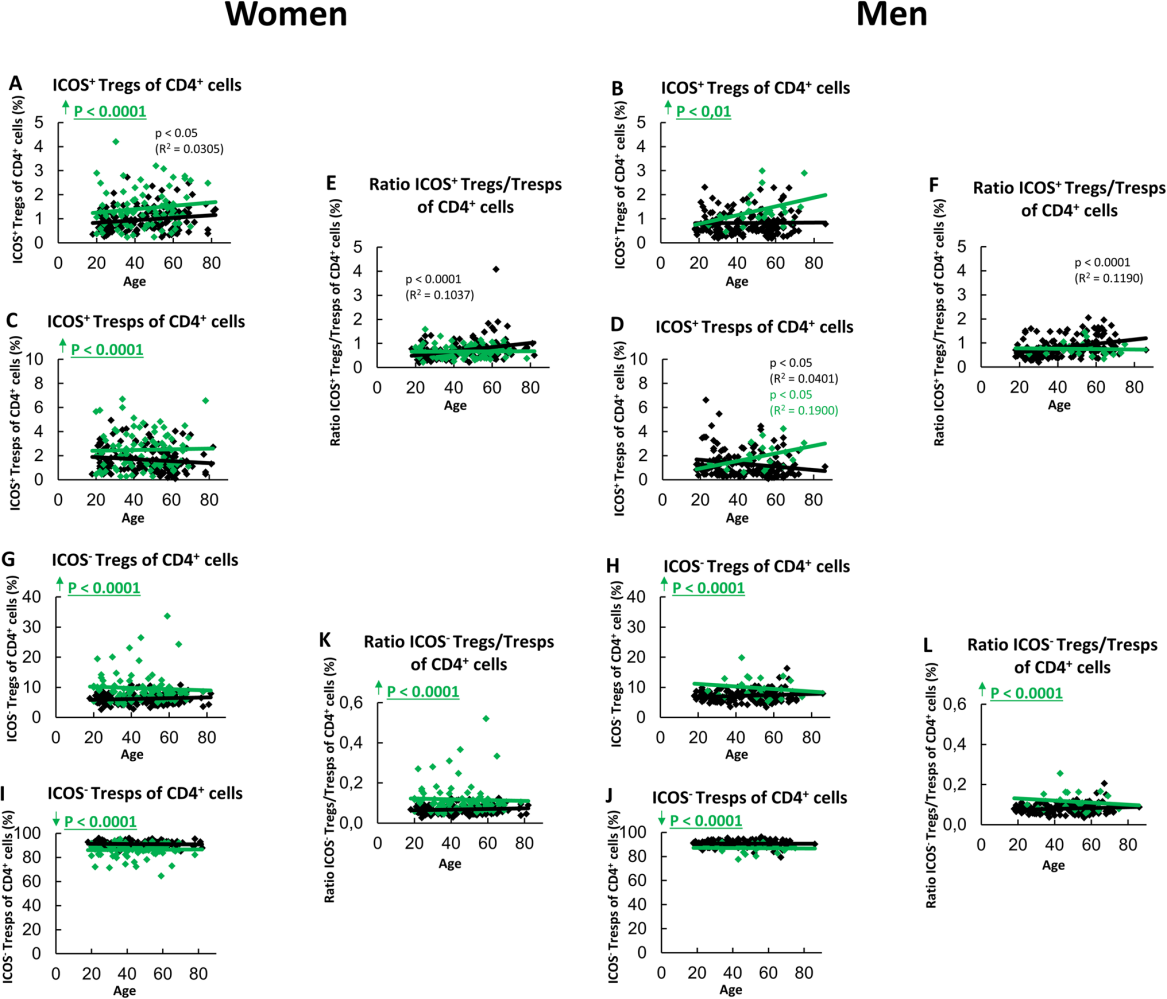
Composition of the CD4^+^ T cell pool with ICOS^+^ and ICOS^−^ Tregs/Tresps and ratio of ICOS^+^/ICOS^−^ Tregs/Tresps in women and men. The percentages of ICOS^+^ Tregs/Tresps within total CD4^+^ T cells in women (**A** and **C**) and men (**B** and **D**) as well as of ICOS^−^ Tregs/Tresps in women (**G** and **I**) and men **(H** and **J**) were estimated depending on age in healthy volunteers () and SLE patients in remission (). The ratios of ICOS^+^ Tregs/Tresps or ICOS^−^ Tregs/Tresps are shown in women (**E** and **K**) and men (**F** and **L**). Significant age-dependent changes are marked by black or green p-values. Significant age-independently increased or decreased percentages/ratios in SLE patients in remission compared to healthy volunteers are marked by an arrow (↑↓) and green underlined *p* values

**Fig. 2:**
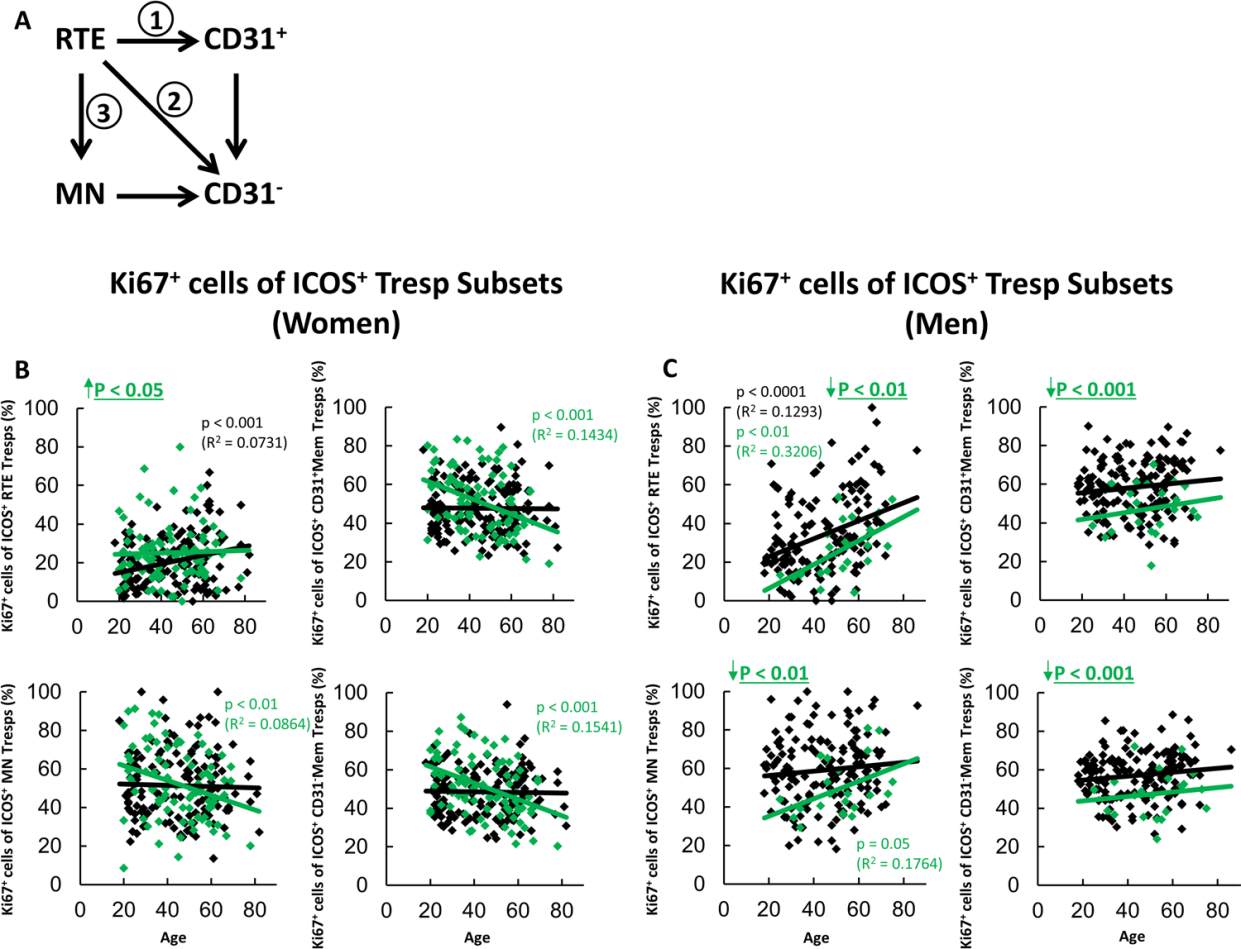
Differentiation pathways of ICOS^+^ RTE Tresps into CD31^−^ memory Tresps and percentages of Ki67^+^ cells within ICOS^+^ Tresp subsets in women and men. Three possible differentiation pathways, via CD31^+^ memory Tresps (pathway 1), via direct proliferation (pathway 2), or via differentiation of resting MN Tresps (pathway 3) are shown in (**A**). The percentages of Ki67^+^ cells within ICOS^+^ RTE, MN, CD31^+^ memory and CD31^−^ memory Tresps in women (**B**) and men (**C**) were estimated depending on age in healthy volunteers () and SLE patients in remission (). Significant age-dependent changes are marked by black or green p-values. Significant age-independently increased or decreased percentages in SLE patients in remission compared to healthy volunteers are marked by an arrow (↑↓) and green underlined p-values

**Fig. 3:**
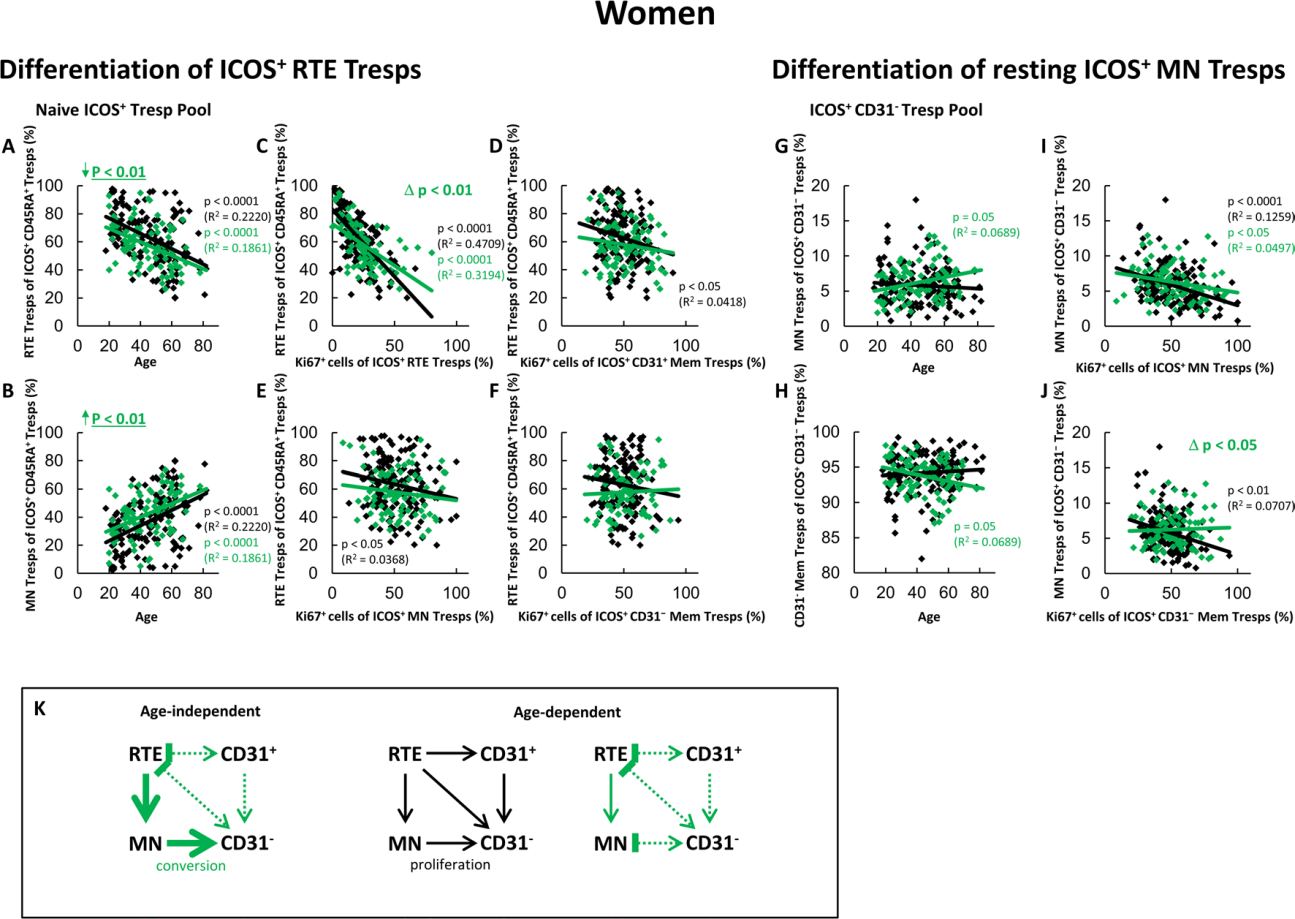
Changing differentiation pathways of ICOS^+^ RTE Tresps in female SLE remission patients compared to healthy volunteers. The percentages of RTE Tresps and MN Tresps within the naïve ICOS^+^ CD45RA^+^ Tresp pool (**A** and **B**) as well as the percentages of MN Tresps and CD31^−^ memory Tresps within the ICOS^+^ CD31^−^ Tresp pool (**G** and **H**) were estimated depending on age in female healthy volunteers () and female SLE patients in remission (). The differentiation of ICOS^+^ RTE Tresps was examined by correlating the percentages of RTE Tresps within total naïve CD45RA^+^ Tresp pool with the percentages of Ki67^+^ cells within total RTE Tresps (**C**), CD31^+^ memory Tresps (**D**), MN Tresps (**E**) and CD31^−^ memory Tresps (**F**). Differentiation of resting MN Tresps was examined by correlating the percentages of MN Tresps within total CD31^−^ Tresp pool with the percentages of Ki67^+^ cells within total MN Tresps (**I**) and CD31^−^ memory Tresps (**J**). Significant correlations are marked by black or green p-values. Significant differences in the regression lines between healthy controls and SLE patients in remission are marked by green Δ p-values. Significant age-independently increased or decreased percentages in female remission patients compared to healthy volunteers are marked by an arrow (↑↓) and green underlined p-values. **K** shows the resulting age-independently increased differentiation compared to healthy controls (green thick arrows), as well as the age-dependent differentiation pathways (color-matched thin arrows). In addition, inhibition of certain pathways by immunosuppressive therapy is shown ()

**Fig. 4:**
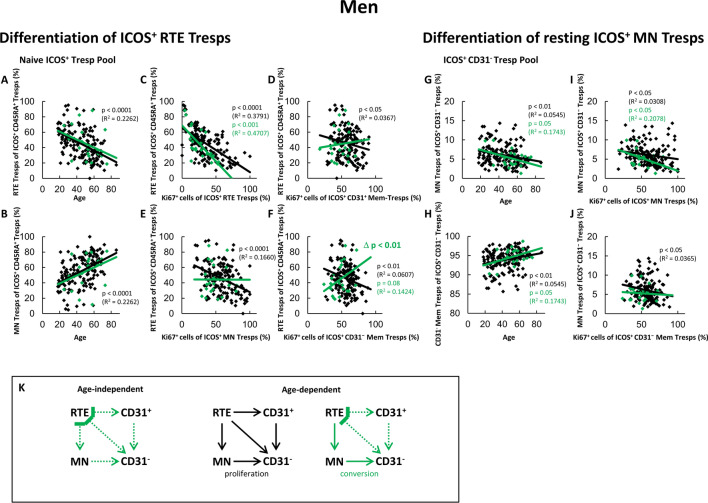
Changing differentiation pathways of ICOS^+^ RTE Tresps in male SLE remission patients compared to healthy volunteers. The percentages of RTE Tresps and MN Tresps within the naïve ICOS^+^ CD45RA^+^ Tresp pool (**A** and **B**) as well as the percentages of MN Tresps and CD31^−^ memory Tresps within the ICOS^+^ CD31^−^ Tresp pool (**G** and **H**) were estimated depending on age in male healthy volunteers () and male SLE patients in remission (). The differentiation of ICOS^+^ RTE Tresps was examined by correlating the percentages of RTE Tresps within total naïve CD45RA^+^ Tresp pool with the percentages of Ki67^+^ cells within total RTE Tresps (**C**), CD31^+^ memory Tresps (**D**), MN Tresps (**E**) and CD31^−^ memory Tresps (**F**). Differentiation of resting MN Tresps was examined by correlating the percentages of MN Tresps within total CD31^−^ Tresp pool with the percentages of Ki67^+^ cells within total MN Tresps (**I**) and CD31^−^ memory Tresps (**J**). Significant correlations are marked by black or green p-values. Significant differences in the regression lines between healthy controls and SLE patients in remission are marked by green Δ p-values. Significant age-independently increased or decreased percentages in male remission patients compared to healthy volunteers are marked by an arrow (↑↓) and green underlined p-values. **K** shows the resulting age-independently not increased differentiation compared to healthy controls (green dotted arrows), as well as the age-dependent differentiation pathways (color-matched thin arrows). In addition, inhibition of certain pathways by immunosuppressive therapy is shown ()

In this study, we used linear regression to correlate the changes in the composition of total CD4^+^ T cells with different subsets such as ICOS^+^ Tregs and ICOS^+^ Tresps, as well as ICOS^−^Tregs and ICOS^−^ Tresps in dependence on age (Fig. 1). This was done using separate models for all patient groups (healthy controls and SLE patients in remission) for both women and men. Thereby, the regression coefficient (slope of the regression line) was statistically compared against zero and the respective *p* value reported. A *p* value < 0.05 was considered significant. The same approach was used for evaluating the changes with age in the percentages of Ki67^+^ cells within ICOS^+^ RTE, MN, CD31^+^ and CD31^−^ memory Tresps (Fig. 2B, C). Likewise, the changes with age in the composition of the naïve ICOS^+^ Tresp cell pools with RTE and MN Tresps, as well as the changes with age in the composition of the ICOS^+^ CD31^−^ Tresp cell pools with MN Tresps and CD31^−^ memory Tresps were examined (Fig. 3 and 4, respectively, A and B as well as G and H).

In order to discover the differentiation pathway of ICOS^+^ RTE Tresps via CD31^+^ memory Tresps (pathway 1) or direct proliferation via MN Tresps (pathway 2), an analogous procedure was chosen by correlating the changes in the percentages of ICOS^+^ RTE Tresps within their naïve CD45RA^+^ Tresps with the Ki67 expression of their respective RTE, MN, CD31^+^ memory, and C31^−^ memory Tresps (Figs. 3 and 4, C—F). Similarly, in order to examine the differentiation of ICOS^+^ resting MN Tresps into ICOS^+^ CD31^−^ memory Tresps (pathway 3), the changes in the percentages of MN Tresps within CD31^−^ Tresps were correlated with the Ki67 expression of their respective MN and CD31^−^ memory Tresps (Figs. 3 and 4, I and J). A p-value < 0.05 was considered significant, where the significance refers to the slope of the regression line. Differences in the differentiation capacity with regard to the respective pathways between patient groups were recognized by statistically comparing the slope of regression lines. A p-value < 0.05 was considered significant.

Age-independent differences between healthy volunteers and SLE patients in remission concerning the above listed Treg/Tresp subsets were examined using multiple regression analysis adjusted for the age variable (centered on the mean of both groups), wherein an interaction term of the age and the patient group was included (Fig. 1, Fig. 2B, C and Figs. 3 and 4, respectively, A and B as well as G and H). Such statistical approaches were calculated separately for women and men. Additionally, in Fig. 1, we used the same approach to find age-dependent or age-independent differences in the composition of the CD4^+^ T cell pool with ICOS^+^ and ICOS^−^ Tregs/Tresps between female and male SLE remission patients. For reasons of clarity, these results are only described in the results section and not presented graphically. For all tests, the software package BiAS for Windows (version 11.12) was used.

## Results

### The percentage of ICOS^+^ Tresps within total CD4^+^ T cells increases with age in male remission patients, but not in women

To investigate whether differences in the composition of the total CD4^+^ T cell pool with ICOS^+^ or ICOS^−^ Tregs/Tresps could be responsible for the higher prevalence of SLE in women compared to men and especially for the characteristic occurrence of this disease in rather young women but older men, we determined the percentages of these Treg/Tresp subsets within the total CD4^+^ T cell pool in healthy controls (Group 1) and SLE patients with low disease activity (Group 2). Thereby, differentially aged women and men were examined separately. Table 1 shows the clinical data of all participants. The time since initial diagnosis was significantly lower in male SLE remission patients compared to female SLE remission patients (*p* < 0.05). The ethnicity of the participants was not considered. However, with the study site being in Heidelberg, Germany, the majority of participants were Caucasians.

**Table 1:** Clinical characteristics of female and male SLE remission patients and healthy controls

	Women		Men	
	Healthy controls	SLE remission patients	Healthy controls	SLE remission patients
	n = 150	n = 83	n = 150	n = 22
Age (years)	45 ± 16	45 ± 14	44 ± 16	52 ± 14
Time since initial diagnosis (months)		185 ± 112		132 ± 97
Renal involvement, *n* (%)		64 (77%)		17 (77%)
SLEDAI		2 ± 2		1 ± 1
ANA titer ≤ 1:1280, *n* (%)		43 (52%)		11 (50%)
DsDNA antibodies ELISA (IU/ml)		66 ± 161		21 ± 16
C3 complement (g/l)		1.1 ± 0.2		1.2 ± 0.2
C4 complement (g/l)		0.2 ± 0.1		0.2 ± 0.1
Erythrocyturia (x μl^−1^)		25 ± 119		6 ± 11
Leukocyturia (× μl^−1^)		18 ± 76		9 ± 23
Serum leucocytes (× nl^−1^)		7 ± 3		7 ± 2
Serum creatinine (mg dl^−1^)		0.9 ± 0.6		1.2 ± 1.0
CKD-EPI GFR (ml min^−1^ (1.73 m^2^)^−1^)		92 ± 32		89 ± 30
Urine-Protein/Urine-creatinine Ratio (g (mol creatinine)^−1^)		63 ± 120		54 ± 90
Medication				
No Medication, *n* (%)		2 (2%)		1 (5%)
Antimalarials, *n* (%)		65 (78%)		15 (68%)
Mycophenolic acid (MPA), *n (*%)		33 (40%)		10 (45%)
Azathioprine (AZA), *n* (%)		20 (24%)		2 (9%)
Glucocorticoids, *n* (%)		49 (59%)		12 (55%)
Glucocorticoid dose (mg d^−1^)		7 ± 8		9 ± 15

*ANA* antinuclear antibodies; *CKD-EPI GFR* chronic kidney disease epidemiology collaboration estimated glomerular filtration rate; *DsDNA antibodies ELISA* double-stranded DNA antibodies enzyme-linked immunosorbent assay; *SLEDAI* SLE disease activity index; *n* number. The data is presented as their mean and standard deviations

In healthy volunteers, we found significantly increasing percentages of ICOS^+^ Tregs with age in women, but not in men (Fig. 1A, B). In contrast, with age, significantly decreasing percentages of ICOS^+^ Tresps were found in men, but not in women (Fig. 1C, D). Nevertheless, an age-dependent increase in the ICOS^+^ Tregs/Tresps ratio could be detected in healthy controls, both in women and men (Fig. 1E, F). The percentages of ICOS^−^ Tregs/Tresps did not change with age, neither in women nor in men (Fig. 1G–J). Accordingly, the ratio of ICOS^−^ Tregs/Tresps remained unchanged depending on age (Fig. 1K, L).

In female SLE remission patients, neither age-dependent changes in the percentages of ICOS^+^ Tregs/Tresps, nor age-dependent changes in the ratio of ICOS^+^ Tregs/Tresps were observed (Fig. 1A, C and E). In male remission patients, no age-dependent changes in the percentages of ICOS^+^ Tregs were detected either (Fig. 1B), but the percentages of ICOS^+^ Tresps increased with age (Fig. 1D). Still, the ratio of ICOS^+^ Tregs/Tresps didn’t change with age (Fig. 1F). Similarly, as in healthy controls, the percentages of ICOS^−^ Tregs, and ICOS^−^ Tresps did not change with age in SLE remission patients, neither in women nor in men (Fig. 1G–J). However, regardless of age, the percentages of ICOS^+^ Tregs were increased compared to healthy controls both in women and men (Fig. 1A, B). The percentages of ICOS^+^ Tresps were significantly increased in women, but not in men (Fig. 1C, D). Nevertheless, the ratio of ICOS^+^ Tregs/Tresps was found to be unchanged in SLE remission patients compared with healthy controls both in women and men (Fig. 1E, F). Accordingly, the percentages of ICOS^−^ Tregs were increased, while those of ICOS^−^ Tresps were decreased compared to healthy controls both in women and men (Fig. 1G–J), which increased the ratio of ICOS^−^ Tregs/Tresps for both sexes (Fig. 1K, L).

By comparing female and male SLE remission patients, no age-dependent or age-independent differences could be detected for ICOS^+^ Tregs and the ratio of ICOS^+^ Tregs/Tresps, as well as for ICOS^−^ Tregs, ICOS^−^ Tresps and the ratio of ICOS^−^ Tregs/Tresps. Regardless of age, only ICOS^+^ Tresps showed lower, but not significantly (*p* = 0.06) reduced percentages in men compared to women. Age-dependently no significance could be ascertained.

These findings may provide a first indication why female SLE patients in remission are more likely to develop active disease, whereas men rather transition into active disease with increasing age. Furthermore, the crucial role of particularly ICOS^+^ Tresps in the development of active disease is shown.

### ICOS^+^ RTE Tresp activation is age-independently strongly suppressed in male SLE remission patients, but not in women

To find an explanation for both the age-independent and age-dependent changes in the percentages of ICOS^+^ Tresps within the CD4^+^ T helper cell pool, we investigated the differentiation of newly released RTE Tresps from the thymus into CD31^−^ memory Tresps. Thereby, the ICOS^+^ Tresp pool may consist of RTE Tresps, resting MN Tresps, CD31^+^ and CD31^−^ memory Tresps, respectively. The differentiation of the RTE Tresps may occur via CD31^+^ memory Tresps into CD31^−^ memory Tresps (pathway 1), or their direct proliferation into CD31^−^ memory Tresps (pathway 2). In addition, the differentiation of resting MN Tresps may contribute to the development of CD31^−^ memory Tresps (pathway 3) (Fig. 2A).

To determine the differentiation pathways by which ICOS^+^ RTE Tresps differentiate into ICOS^+^ CD31^−^ memory Tresps, we determined the percentages of Ki67^+^ T cells within RTE Tresps, MN Tresps, CD31^+^ memory Tresps, and CD31^−^ memory Tresps in healthy controls of different ages compared with SLE patients with low disease activity. Figure 2B and C shows that in healthy controls, the percentages of Ki67^+^ T cells within RTE Tresps increase significantly with age, in both women and men, while within the other Tresp subsets their percentages remain relatively constant.

In female SLE patients in remission, a significant age-independent increase of Ki67^+^ T cells within RTE Tresps was observed, which could not be detected for any other subset, due to the fact that the percentage of Ki67^+^ T cells decreased significantly with age within these subsets (Fig. 2B). In contrast, in male SLE patients in remission, the percentage of Ki67^+^ T cells was age-independently significantly decreased in all Tresp subsets. However, with age, their percentage increased significantly within RTE Tresps and MN Tresps (Fig. 2C).

Such findings may propose that, in healthy controls, with age, an increasing activation of ICOS^+^ RTE Tresps favors their balanced differentiation via pathway 1 and pathway 2 or 3 into CD31^−^ memory Tresps in both women and men. In female SLE patients in remission, the insufficient immunosuppression seems to be associated with excessive age-independent activation of the RTE Tresps, whereby their age-dependent differentiation via pathway 1 and pathway 2 or 3 may be suppressed, so that the percentage of ICOS^+^ Tresps does not increase within total CD4^+^ T cells in women (Fig. 1C). In contrast, in men, the immunosuppressive therapy was associated with a strong age-independent suppression of the activation of RTE Tresps, so that their age-dependent activation and differentiation via pathway 2 or 3 may be favored. Presumably, this favors the significant increase of ICOS^+^ Tresps within total CD4^+^ T cells with age (Fig. 1D).

### In female remission patients, there is an age-independently increased differentiation of ICOS^+^ RTE Tresps into CD31^−^ memory Tresps via conversion of resting MN Tresps

To identify the differentiation pathways more accurately, we correlated the changes in RTE Tresps in the naïve Tresp pool with the proportion of Ki67^+^ T cells within each of the four Tresp subsets. Furthermore, we correlated the changes of resting MN Tresps in the CD31^−^ Tresp pool with the proportion of Ki67^+^ T cells within the MN Tresp pool and the CD31^−^ memory Tresp pool. Figure 3 shows the results obtained for the differentiation of ICOS^+^ RTE Tresps and resting MN Tresps in women for both patient groups.

For healthy women, we found significantly decreasing percentages of RTE Tresps and complementary increasing percentages of MN Tresps within the naïve ICOS^+^ Tresp pool with age (Fig. 3A, B) and ascertained a significant negative correlation between the percentages of RTE Tresps within total naïve Tresps and the percentages of Ki67^+^ cells within total RTE Tresps (Fig. 3C). A similar significant correlation between the percentages of RTE Tresps within naïve Tresps and the percentages of Ki67^+^ cells within both CD31^+^ memory Tresps and resting MN Tresps was detected (Fig. 3D, E). Therefore, it seems that with age, RTE Tresps are activated and differentiate via both CD31^+^ memory Tresps (pathway 1) and direct proliferation via MN Tresps into CD31^−^ memory Tresps (pathway 2). A significant correlation between the percentages of RTE Tresps within naïve Tresps and the percentages of Ki67^+^ cells within CD31^−^ memory Tresps was not detected (Fig. 3F). Furthermore, we found a significant negative correlation between the percentages of resting MN Tresps within total CD31^−^ Tresps and their percentages of Ki67^+^ cells (Fig. 3I), as well as a significant negative correlation between the percentages of resting MN Tresps within total CD31^−^ Tresps and the percentages of Ki67^+^ cells within CD31^−^ memory Tresps (Fig. 3J). As the percentages of resting MN Tresps within total CD31^−^ Tresps did not change with age (Fig. 3G, H), this means that there is a balanced regulated proliferation of resting MN Tresps into CD31^−^ memory Tresps, when too few resting MN Tresps are produced, so that the Ki67 activity of the CD31^−^ memory pool is increased. Therefore, with age, a parallel differentiation of RTE Tresps via all three pathways (pathway 1, 2 and 3) seems to occur in healthy women (Fig. 3K).

In female remission patients, a significantly increased age-independent shift in the naïve ICOS^+^ Tresp pool towards resting MN Tresps compared to healthy women (Fig. 3A, B) shows the increased age-independent differentiation of these cells. This is supported by the increased differentiation capacity compared to healthy women, as seen by the significant difference in the slopes of the regression lines in Fig. 3C. Since no correlations could be ascertained between the percentages of RTE Tresps within naïve Tresps and the percentages of Ki67^+^ cells within both CD31^+^ memory Tresps and resting MN Tresps (Fig. 3D, E), an inhibition of pathway 1 and 2 by the immunosuppressive therapy may be assumed. Despite the age-independent shift in Fig. 3A and B no shift was ascertained in Fig. 3G and H, but a significant negative correlation between the percentages of resting MN Tresps and their Ki67 activation (Fig. 3I). In contrast to healthy controls, no correlation between the percentages of resting MN Tresps within total CD31^−^ Tresps and the percentages of Ki67^+^ cells within CD31^−^ memory Tresps was found, but a significant difference in the slopes of the regression lines (Fig. 3J). These findings suggest that, age-independently, an increased differentiation via conversion of resting MN Tresps into CD31^−^ memory Tresps takes place (pathway 3, Fig. 3K). With age, only a significant shift towards resting MN Tresps could be detected in Fig. 3A and B, as well as an almost significant increase of the percentages of resting MN Tresps within total CD31^−^ Tresps (Fig. 3G). This suggests that, with age, merely a production of resting MN Tresps occurs, which do not differentiate into CD31^−^ memory Tresps. This may explain the decreasing percentages of Ki67^+^ cells within all ICOS^+^ Tresp subsets except RTE Tresps (Fig. 2B), confirming a strong suppression of the age-dependent differentiation in female SLE remission patients, as well as the age-independently increased percentages of ICOS^+^ Tresps within the total CD4^+^ T helper cell pool (Fig. 1C).

### In male remission patients, there is an age-dependent differentiation of ICOS^+^ RTE Tresps into CD31^−^ memory Tresps via conversion of resting MN Tresps

Figure 4 shows the age-dependent differentiation of ICOS^+^ RTE Tresps and resting MN Tresps in healthy men together with its age-independent and age-dependent changes in SLE patients in remission. For healthy controls, we found significantly decreasing percentages of ICOS^+^ RTE Tresps and complementary increasing percentages of MN Tresps within the naïve Tresp pool (Fig. 4A, B). A negative correlation between the percentages of RTE Tresps within naïve Tresp pool and the percentages of Ki67^+^ cells within RTE Tresps was also ascertained (Fig. 4C). Furthermore, a significant negative correlation between the percentages of RTE Tresps within the naïve Tresp pool and the percentages of Ki67^+^ cells within both CD31^+^ memory Tresps and MN Tresps could be found (Fig. 4D, E). This shows that, with age, the ICOS^+^ RTE Tresps are activated and differentiate via CD31^+^ memory Tresps and via direct proliferation into CD31^−^ memory Tresps (pathway 1 and 2, Fig. 4K). Considering the differentiation of ICOS^+^ resting MN Tresps, with age, a significant shift in the CD31^−^ Tresp pool towards CD31^−^ memory Tresps could be detected (Fig. 4G, H). In addition, a negative correlation could be ascertained between the percentages of MN Tresps within total CD31^−^ Tresps and the percentages of Ki67^+^ cells within both MN Tresps and CD31^−^ memory Tresps (Fig. 4I, J). Also, a significant negative correlation between the percentages of RTE Tresps within naïve Tresps and the percentages of Ki67^+^ cells within CD31^−^ memory Tresps was detected (Fig. 4F), overall suggesting an additional regulated proliferation of resting MN Tresps into CD31^−^ memory Tresps with age (pathway 3, Fig. 4K).

In male remission patients, no age-independent shift was detected in either the naïve Tresp pool or the total CD31^−^ Tresp pool compared with healthy controls (Fig. 4A–B and G–H), which is not indicative of increased differentiation (Fig. 4K). With age, although not significant, a shift in the naïve Tresp pool towards MN Tresps can be assumed by the slopes of the regression lines (Fig. 4A, B). Additionally, the significant negative correlation in Fig. 4C shows an age-dependent RTE activation and differentiation. No correlations were detected in Fig. 4D and E, indicating a suppression of pathway 1 and 2 by the immunosuppressive therapy. However, an almost significant shift in the total CD31^−^ pool towards CD31^−^ memory Tresps was ascertained (Fig. 4G and H), together with a significant negative correlation between the percentages of MN Tresps within total CD31^−^ Tresps and their percentages of Ki67^+^ cells (Fig. 4I). Considering the almost significant positive correlation and the significant difference in the slopes of the regression lines compared to healthy controls in Fig. 4F, as well as the absence of a negative correlation in Fig. 4J, a differentiation via conversion of resting MN Tresps into CD31^−^ memory Tresps seems to occur with age (pathway 3, Fig. 4K). This may explain the age-dependent increase of the percentages of Ki67^+^ T cells within RTE Tresps and MN Tresps (Fig. 2C) as well as the age-dependent increase of ICOS^+^ Tresps within the CD4^+^ T helper cell pool (Fig. 1D).

## Discussion

The pathophysiologic mechanisms responsible for the sex- and age-specific occurrence of SLE are still unclear. Hormones, such as estrogens, were shown to influence thymic output as well as the development and function of CD4^+^ T cells [44–46]. Thereby, differences in hormonally influenced differentiation of opposing T cell populations like Tregs and Tresps seem to play a critical role. In this context, distinct subsets of these T cell populations may be affected differentially by immunosuppression. Our previous study, examining mixed collectives of both sexes already identified highly proliferative ICOS^+^ Tregs/Tresps as key populations, whose ratio showed a characteristic age-independent shift in favor of ICOS^+^ Tresps when the disease activity changed from remission to active disease. In contrast, the ratio of ICOS^−^ Tregs/Tresps was changed in favor of ICOS^−^ Tregs, suggesting that rather ICOS^+^ Tresps than ICOS^−^ Tresps may be responsible for disease progression [32]. In the present study, we identified ICOS^+^ Tresps as the only T cell population showing differences between female and male remission patients by comparing the composition of the CD4^+^ T helper cell pool separately for both sexes. Thereby, it became apparent that in female SLE remission patients the percentage of ICOS^+^ Tresps was age-independently increased, while in men only an age-dependently increasing percentage of ICOS^+^ Tresps was found. These findings may correlate with the clinical presentation of this disease, which rather affects women than men who are more likely to be affected with increasing age.

Since such data suggest sex-specific differences in both the age-independent and age-dependent differentiation of ICOS^+^ Tresps, we identified the differentiation pathways of ICOS^+^ RTEs that occur in healthy subjects compared to SLE patients in remission, separately for women and men. For a long time, it was assumed that RTEs undergo post-thymic selection and proliferation producing long-living MN cells which maintain the size of the naïve T cell pool but exhibit diminished TCR specificity, while cytokine-driven homeostatic proliferation expands the RTE pool to preserve T cell diversity [47–50]. To date it remains unknown which of these naïve T cell populations is preferentially used to replenish the memory T cell pool, either in normal aging, or in specific diseases. Murine models demonstrated that only in case of special need, such as in neonates or lymphopenic adults, RTEs instead of MNs are incorporated into the T cell pool [51]. Data, recently collected in healthy subjects and SLE patients, rather propose successive differentiation pathways with a finely tuned conversion or proliferation of both subsets during normal aging or disease progression, which may be influenced by immunosuppression and differ in women and men [32].

In this study, we found strong sex-specific differences regarding the effect of immunosuppression on ICOS^+^ Tresp activation and differentiation, which proved to be very effective in men, but which was less effective in women, irrespective of age. As a consequence, age-dependent activation and differentiation of ICOS^+^ resting MN Tresps was enhanced in men, whereas in women, age-dependent activation and differentiation was completely inhibited via all three pathways. The more detailed investigation of the differentiation pathways in SLE remission patients compared to healthy controls confirmed these assumptions. Therefore, the processes described here may explain the age-independent increase of ICOS^+^ Tresps within total CD4^+^ T cells in women, but not men, while with age ICOS^+^ Tresps increased in men, but not in women. Further prospective studies may be necessary to confirm these findings, whereby experimental approaches in which ICOS^+^ RTE Tresps of both sexes are stimulated in vitro under the influence of immunosuppressive agents could also help to demonstrate the differential differentiation of ICOS^+^ Tresps in women and men.

Because both ICOS^+^ Tregs and especially ICOS^+^ Tresps appear to play a critical role in the onset of active disease, further studies are needed to further characterize the specific ICOS^+^ Treg/Tresp subsets involved in the pathogenesis of SLE. It is known that ICOS is predominantly expressed on both follicular and regulatory follicular T helper cells (Tfh and Tfr cells), which act antagonistically to regulate quantity and quality of humoral immunity. Several studies have shown an increased frequency of circulating Tfh cells and therefore decreased Tfr/Tfh ratio in SLE patients compared to healthy controls with the Tfr/Tfh ratio correlating with disease activity [52–54]. On the other hand, contradicting results with increased circulating Tfr/Tfh ratios have also been reported [55]. Recently, a new peripheral CD4^+^ T cell subset, termed peripheral helper T (Tph) cells, which has the capacity to promote B cell responses and antibody production was reported [42]. Similar to Tfh cells, Tph cells express ICOS as well as PD-1 and produce IL-21, but lack CXCR5 expression. These cells were shown to be expanded in the periphery of patients with various antibody-mediated autoimmune diseases [56], playing an important role in SLE development [57, 58]. While Tfr cells are known to arise from FoxP3^+^ precursors from the thymus [59], both Tfh and Tph are thought to develop from naive CD4^+^ T cells under the influence of specific cytokines and other mediators [60]. Therefore, it seems more likely that the ICOS^+^ Tresps examined in this study presumably represent thymus-derived, natural Th17 cells, which also express ICOS and were shown to be critically involved in the pathogenesis of SLE [61, 62]. Although this can only be suspected, our data suggest that this highly proliferative ICOS^+^ Tresp subset is responsible for the differential development of high disease activity in women and men. It appears that immunosuppression inhibits individual differentiation pathways (pathways 1 and 2) equally in males and females, while the resulting increased differentiation of ICOS^+^ resting MN Tresps (pathway 3) is still sufficiently suppressed in males, but not in females. This may affect the age-dependent differentiation of resting MN Tresps differently in men and women.

Our calculations and the resulting interpretations may have limitations such as low cell numbers in the experimental approaches, especially for ICOS^+^ T cells, and the lack of functional experiments. It is also to be considered that although most participants were Caucasians, the ethnicity was not specifically inquired. Since the prevalence of SLE is dependent on race, for example being higher in Asians and Hispanics compared to Caucasians [63], there may be an uneven racial distribution between female and male healthy control groups and SLE remission patient groups as a confounding factor. Furthermore, low patient numbers, especially in the male diseased patient group, as well as unequal numbers of subjects in the different groups may reduce the significance of our findings. Therefore, further studies are needed, most likely involving multiple study sites, to confirm our results.

In summary, we show that the immunosuppressive therapy used in SLE patients may have different effects on the differentiation of ICOS^+^ Tresps in women and men. While in female remission patients an age-independent insufficient immunosuppression induces an increased suppression with age, in male remission patients, an age-independent strong suppression leads to a reduced effect of immunosuppression with age. These findings may explain the occurrence of high disease activity preferentially in women and older men and may provide a basis for exploring a sex- and age-specific therapy for SLE.

## Supplementary Information

Below is the link to the electronic supplementary material.

Supplementary file1 (DOCX 322 kb)

## Data Availability

Original data on flow cytometric determinations are available from the corresponding author upon request.

## Abbreviations

ACR: American College of Rheumatology
Anti-dsDNA: Anti-double-stranded DNA
CD31^−^ memory Tregs/Tresps: CD45RA^−^CD31^−^ memory Tregs/Tresps
CD31^+^ memory Tregs/Tresps: CD45RA^−^CD31^+^ memory Tregs/Tresps
ICOS: Inducible costimulatory molecule
MN Tregs/Tresps: CD45RA^+^CD31^−^ mature naïve Tregs/Tresps
RTE: Recent thymic emigrant
SLE: Systemic lupus erythematosus
SLEDAI: Systemic lupus erythematosus disease activity index
Tregs: Regulatory CD4^+^ T cells
Tresps: Non-regulatory responder CD4^+^ T cells
