# Supplementary material for: Sex-specific differences in ICOS+ T helper cell differentiation in systemic lupus erythematosus patients with low disease activity
Source: Clin Exp Med. 2024 Mar 1;24(1):47. doi: 10.1007/s10238-024-01307-1 (PMC10907489; doi:10.1007/s10238-024-01307-1)
Supplement: Supplementary file 1 — Supplementary file1 (DOCX 322 kb) [file 10238_2024_1307_MOESM1_ESM.docx]

**Supplementary Information**

**
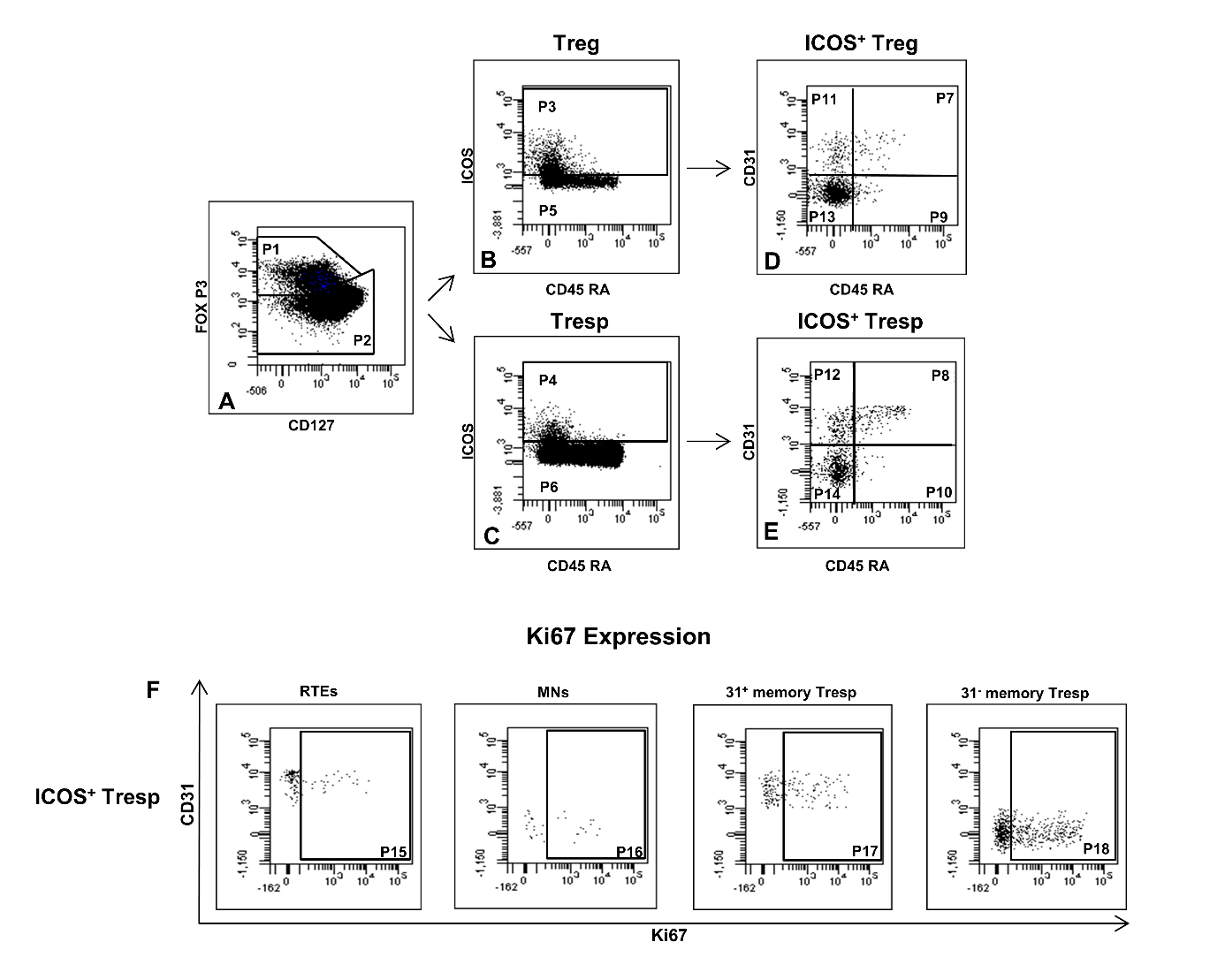
**

**Supplementary Fig. 1**: Gating strategy. Positively isolated CD4^+^ T helper cells were divided into CD4^+^CD127^low+/-^FoxP3^+^ Tregs (P1) and CD4^+^CD127^+^FoxP3^-^ Tresps (P2) by gating fluorescence intensity of FoxP3 versus CD127 (**A**). ICOS^+^ Tregs/Tresps (P3, P4) and ICOS^-^ Tregs/Tresps (P5, P6) were gated by fluorescence activity of ICOS versus CD45RA (**B and C**). The percentages of ICOS^+^ RTE Tregs/Tresps (P7, P8), MN Tregs/Tresps (P9, P10), CD31^+^ memory Tregs/Tresps (P11, P12) and CD31^-^ memory Tregs/Tresps (P13, P14) were estimated by analyzing ICOS^+^ Tregs (P3), (**D**) and ICOS^+^ Tresps (P4), (**E**) for its fluorescence intensity of CD31 versus CD45RA. The Ki67 expression of ICOS^+^ RTE, MN, CD31^+^ memory and CD31^-^ memory Tresps (P15 - P18) were estimated by analyzing the fluorescence intensity of CD31 versus Ki67 (**F**).
